# Research on the impact of health empowerment on self-management in older patients with chronic disease: based on chain mediating effect analysis

**DOI:** 10.3389/fpubh.2025.1681312

**Published:** 2025-10-21

**Authors:** Tiemei Wang, Shiqing Huang, Jinxia Liu, Yan Liu

**Affiliations:** ^1^Outpatient Department, The Third People’s Hospital of Chengdu and The Affiliated Hospital of Southwest Jiaotong University, Chengdu, Sichuan, China; ^2^Jinfeng Laboratory, Chongqing, China; ^3^Intensive Care Unit, The Third People’s Hospital of Chengdu and The Affiliated Hospital of Southwest Jiaotong University, Chengdu, Sichuan, China; ^4^West China School of Public Health and West China Fourth Hospital, West China Lecheng Hospital, Sichuan University, Chengdu, Sichuan, China

**Keywords:** older adults, chronic diseases, self-management, health empowerment, chronic disease resource utilization, self-efficacy

## Abstract

**Objective:**

This study investigates the mediating effect of chronic disease resource utilization and self-efficacy on the relationship between health empowerment and self-management in older adults with chronic diseases.

**Methods:**

A convenience sampling method was used to select 826 older adult patients with chronic diseases from 12 tertiary hospitals in Sichuan Province. General demographic information was collected using a questionnaire, and self-management, health empowerment, chronic disease resource utilization, and self-efficacy were assessed using standardized scales. Data were analyzed using SPSS 25.0, and the PROCESS macro was employed to test the mediating effect of chronic disease resource utilization and self-efficacy.

**Results:**

The mean score for self-management was 40.89 ± 11.71, for health empowerment was 22.07 ± 7.13, for chronic disease resource utilization was 67.02 ± 10.65, and for self-efficacy was 21.25 ± 4.77. Health empowerment was positively correlated with chronic disease resource utilization (*r* = 0.87, *p* < 0.001) and self-efficacy (*r* = 0.75, *p* < 0.001), as well as self-management (*r* = 0.73, *p* < 0.001). Chronic disease resource utilization was positively correlated with self-efficacy (*r* = 0.68, *p* < 0.001), and self-management (*r* = 0.86, *p* < 0.001). Self-efficacy was positively correlated with self-management (*r* = 0.68, *p* < 0.001). Health empowerment can directly and positively affect self-management, with an estimated value of 0.174, accounting for 37.6% of the total effect, and it can influence self-management through chronic disease resource utilization, with an estimated value of 0.123, accounting for 26.6% of the total effect. Health empowerment can influence self-management through self-efficacy, with an estimated value of 0.103, accounting for 22.2% of the total effect, and it can also influence self-management through the chain mediating effect of chronic disease resource utilization and self-efficacy, with an estimated value of 0.063, accounting for 13.6% of the total effect.

**Conclusion:**

Health empowerment can directly affect the self-management of older adults patients with chronic diseases, and it can also influence it indirectly through the utilization of chronic disease resources and self-efficacy. The utilization of chronic disease resources and self-efficacy play a mediating role between health empowerment and self-management. Healthcare providers can improve health empowerment and self-management in older adult patients through effective interventions to enhance chronic disease resource utilization and self-efficacy.

## 1 Introduction

The health crisis triggered by an aging population has become a major public health issue worldwide that urgently needs to be addressed. In our country, surveys show that one fifth of the population are older adults, 78% of whom suffer from chronic diseases, with the vast majority having two or more. It is estimated that, by 2030, the proportion of older population in China will reach 27%, with the number of those with chronic diseases set to triple. The disease burden caused by chronic diseases is expected to increase by at least 40% (1). Consequently, there is an urgent necessity for society to implement effective measures that will promote healthier aging and reduce the burden of chronic diseases on individuals and the medical system (2). The enhancement of self-management abilities during the course of a disease has become a significant public health concern in the context of global aging (3). Self-management has been defined as an ability and process that an individual uses to control their disease (4). Self-management is a pivotal component in the management of chronic diseases, facilitating better adjustment to the illness and reducing the likelihood of complications (5). Self-management is crucial for controlling chronic. A number of studies have demonstrated an association between health empowerment (6, 7), chronic disease resource utilization (8, 9), self-efficacy (10, 11) and self-management in certain chronic conditions. We observed that chronic disease resource utilization and self-efficacy may act as pivotal intermediary variables between health empowerment and self-management among patients with chronic diseases. This notion is supported by the Health Empowerment Theory (12), the health of an individual is influenced by a multitude of factors, including but not limited to the quality of healthcare services provided by the healthcare system. Additionally, the psychological state of the individual, their lifestyle choices, and the social environment they inhabit play a significant role in determining their overall health status. It can thus be concluded that, in cases where patients have sufficient personal resources, such as self-building ability (self-efficacy) and social resources, including social support and social services (resource utilization), they are more likely to adopt active self-management strategies when facing chronic diseases. A large number of research has demonstrated the interrelationship between self-efficacy, health empowerment and chronic diseases in the older adult population. However, there is a paucity of studies that have investigated the relationship between health empowerment, chronic disease resource utilization, self-efficacy and self-management. To the best of our knowledge, no research has hitherto investigated the relationship between these variables among older adult patients with chronic diseases in south-west China. It is imperative that this research deficit be addressed, given the high prevalence of chronic diseases and the significant burden that chronic diseases place on individuals and the medical system in this population. The identification of these relationships is of crucial importance for the development of appropriate interventions aimed at enhancing self-management among older adult patients suffering from chronic diseases. The present study aims to investigate the mediating effect of chronic disease resource utilization and self-efficacy on the relationship between health empowerment and self-management in older adult patients with chronic diseases. The findings will provide a basis for intervention measures in this population.

## 2 Materials and methods

### 2.1 Study participants

Convenience sampling was employed to select patients aged 60 and above who were admitted to the internal medicine wards of 12 tertiary hospitals in Sichuan Province, from June to October 2022. A WeChat questionnaire was administered to a sample of older individuals who use smartphones with the aim of conducting an on-site network survey. Conversely, for older individuals who did not possess smartphones or who had no mobile phones, a paper version of the questionnaire was distributed for the on-site survey. The inclusion criteria for participation in this study are as follows: (1) be aged 60 and over; (2) have been diagnosed with one or more chronic diseases; (3) have a certain level of reading comprehension; and (4) provide informed consent and participate in the study voluntarily. Participants were excluded from the study if they met any of the following criteria: (1) experienced communication difficulties as a result of deafness, speech impairment; (2) cognitive impairment.

### 2.2 Study methods

#### 2.2.1 Research tools

##### 2.2.1.1 General demographic questionnaire

A self-designed questionnaire was employed to collate data pertaining to the patients’ gender, age, marital status, residence, educational level, monthly personal income, monthly per capita household income, living arrangement, times of hospitalizations.

##### 2.2.1.2 Chronic illness resources scale

The CIRS, originally developed by Glasgow et al. (13), was subsequently translated into Chinese by Lv and Zhang (14). The scale compromises 7 dimensions: medical and health teams (3 items), family and friends (3 items), personal responses (3 items), neighborhood communities (4 items), media policies (3 items), organizational structures (2 items) and working environments (as the respondents were retired, the scores pertaining to the working environment were not incorporated in the mean score of the overall scale). Each item is scored on a 4-point Likert scale, ranging from “never” to “always,” with scores ranging from 1 to 4. The total score on the scale ranges from 21 to 84 points, with higher score indicating a better situation for patients in terms of obtaining and utilizing resources. This Chinese version of this scale has been employed extensively across diverse contexts and has demonstrated robust psychometric properties.

##### 2.2.1.3 General self-efficacy scale

The GSES, was conducted by Li et al. (15), and was subsequently translated into Chinese by Yang et al. (16). The scale comprises 10 items that assess an individual’s confidence in their ability to overcome setbacks and difficulties. The evaluation of each item is undertaken employing a 4-point Likert scale, ranging from “completely incorrect” to “completely correct,” with scores ranging from 1 to 4. The total score of the scale is derived by summing the scores for the 10 items. The total score on the scale ranges from 10 to 40 points, with higher scores indicating greater self-efficacy. The Chinese version of this scale has been employed extensively across a range of contexts and has demonstrated robust psychometric properties.

##### 2.2.1.4 Simplified elders health empowerment scale

The SEHES, originally developed by Cheng et al. (17), was subsequently translated into Chinese by Caruso et al. (18), the scale under consideration comprises a total of eight items, the purpose of which is to assess health empowerment in older adults suffering from chronic illnesses. Each item is scored on a 5-point Likert scale, ranging from “strongly disagree” to “strongly agree,” with scores ranging from 1 to 5. The summation of the scores for the eight items in question constitutes the scale’s total score. The total score on the scale ranges from 8 to 40 points, with higher scores indicating higher levels of empowerment. The Chinese version of this scale has been employed extensively across a range of contexts and has demonstrated robust psychometric properties.

##### 2.2.1.5 Chronic disease self-management behavior measuring scale

The CDSMBMS, originally developed by Orig et al. (19), was subsequently translated into Chinese by Cui et al. (20), the scale comprises a total of three dimensions: Exercise (6 items), cognitive symptom management (6 items) and communication with doctors (3 items). The aim is to assess the self-management ability of older adults with chronic diseases. Each item is scored on a 5-point Likert scale, ranging from “never” to “always,” with scores ranging from 1 to 5. The summation of the scores for the 15 items in question constitutes the scale’s total score. The total score on the scale ranges from 15 to 75 points, with higher scores indicating higher levels of self-management. The Chinese version of this scale has been employed extensively across a range of contexts and has demonstrated robust psychometric properties.

#### 2.2.2 Data collection and quality control methods

In accordance with the established standardized instructions, the investigators elucidated the purpose, content, requirements and confidentiality of the survey to the patients. Following consent, the questionnaires were given to the patients. The investigators subsequently reviewed the completed questionnaires on-site and collated them. Two individuals were responsible for the organization, verification and entry of the data, while any invalid questionnaires were excluded from the study. A total of 850 questionnaires were distributed, yielding a 97.18% return rate of 826 completed questionnaires.

#### 2.2.3 Statistical analysis

The statistical analysis was conducted using SPSS 25.0 software. In accordance with the principles of descriptive statistics, the continuous variables were characterized by means and standard deviations. Independent samples *t*-tests and one-way analyses of variance (ANOVAs) were employed to compare the means between the groups in question. Where discrepancies existed in the number of items included in the dimensions under consideration, the scores were normalized and subsequently compared. Pearson’s correlation coefficient was used to examine the relationships between the variables. The PROCESS macro (Model 6) was used to assess the mediating effect of self-efficacy. Bootstrap resampling with 5,000 samples was conducted to estimate 95% confidence intervals for various effects.

#### 2.2.4 Common method bias test

To control for common method bias, this study used anonymous surveys with reverse item settings and items presented in different contexts (21). A confirmatory factor analysis was also conducted using AMOS 25.0 software. The model fit indices were as follows: The model fit indices indicated an inadequate fit, with a χ^2^/df ratio of 4.22, an RMSEA of 0.92, a CFI of 0.83, and an NFI of 0.77. Consequently, the likelihood of substantial common method bias was effectively eliminated in this study.

## 3 Results

### 3.1 Characteristics of participants

Table 1 presents a summary of the demographic characteristics of the participants. The sample was predominantly composed of male, with the majority of respondents being married and aged between 60 and 75. Of all respondents, 489 were come from town and 309 lived with their spouse, while 514 had completed junior high school or less. The proportion of the population with a monthly income between 3,001 and 4,000 yuan is 38.01%. A similar situation is observed in the monthly per capita household income. Significant differences in self-management score between gender, age and across residence groups, but there is no significant differences across the other study variables.

**Table 1:** Univariate analysis of scores on the self-management assessment scale for older adults patients with chronic diseases (*n* = 826).

Variable	Group	Frequency	Score (Mean ± SD)	*t*/*F* value	*p*-value
Gender	Male	472	41.65 ± 10.23	211.063^a^	0.041
	Female	354	40.06 ± 9.33		
Age	60 ~ 75	306	45.54 ± 11.93	4.517^b^	0.002
	76 ~ 85	254	43.19 ± 12.34		
	86 ~ 95	258	44.26 ± 11.25		
	>95	8	47.18 ± 10.23		
Marital status	Unmarried	15	43.26 ± 11.65	2.128^b^	0.516
	Married	509	40.29 ± 11.38		
	Divorced	113	38.69 ± 9.59		
	Widowed	189	35.27 ± 10.79		
Residence	Town	489	41.11 ± 9.82	5.206^a^	0.001
	Rural areas	337	45.63 ± 10.23		
Education level	Primary school and below	296	42.25 ± 10.34	4.758^b^	0.589
	Junior high school	218	41.49 ± 15.21		
	High school or technical school	189	39.98 ± 12.90		
	College or undergraduate	98	37.58 ± 9.97		
	Postgraduate and above	25	44.53 ± 12.07		
Monthly personal income	Below 2,000	125	47.82 ± 10.23	0.592^b^	0.403
	2,001–3,000	263	43.19 ± 10.24		
	3,001–4,000	314	45.12 ± 10.54		
	Above 4,000	124	48.32 ± 13.41		
Monthly per capita household income	Below 3,000	115	35.45 ± 9.08	0.751^b^	0.404
	3,001–4,000	207	42.17 ± 10.93		
	4,001–5,000	185	41.42 ± 10.54		
	Above 5,000	103	43.58 ± 12.10		
Living arrangement	Spouse and children	218	38.88 ± 9.58	4.952^b^	0.058
	Spouse	309	50.20 ± 11.42		
	Children	116	42.41 ± 9.78		
	Living alone	113	40.55 ± 11.21		
	Other	70	38.39 ± 10.45		
Hospitalization times	Once	201	42.76 ± 12.33	598.000^b^	0.061
	Twice	363	44.14 ± 9.57		
	More than three times	262	39.357 ± 10.25		

a Univariate analysis performed using *t*-test.

b Univariate analysis performed using analysis of variance.

Firstly, the self-management level of older female adults has been found to exceed that of their male counterparts. This phenomenon may be attributed to the greater familial responsibilities often shouldered by women, compelling them to competently oversee their own health in order to sustain physical and mental equilibrium. Furthermore, in comparison to males, females exhibit heightened sensitivity with regard to emotional matters and demonstrate a greater propensity to attend to and prioritize diseases. Conversely, men, as the primary income generator within the family unit, are confronted with dual pressures stemming from their professional and familial responsibilities. This dual burden often results in men neglecting their health management, thereby increasing their risk of developing and potentially fatal diseases. Secondly, regarding age difference, two potential explanations for this phenomenon are posited. On the one hand, in terms of cognitive ability, younger seniors usually maintain better cognitive functions than older ones, including memory, learning ability, comprehension and executive functions (such as planning and decision-making). This is of critical importance for the comprehension of intricate medical directives, the recollection of temporal parameters and pharmaceutical dosages, and the monitoring of symptoms. However, the older population is more susceptible to cognitive decline, including mild cognitive impairment and dementia, which directly impairs their capacity for independent living. Conversely, with respect to physical functions, younger older adults generally exhibit superior physical strength, vision and hearing than older population. They are able to access healthcare facilities with greater ease, comprehend the instructions provided with pharmaceuticals, and utilize medical apparatus such as blood glucose meters and blood pressure monitors. However, the older adults may encounter challenges in completing these daily management tasks due to their deteriorating physical capabilities, the presence of comorbidities (the existence of multiple diseases), and fatigue. Thirdly, regarding residence, the present study found that older adults suffering from chronic diseases and residing in urban areas exhibited higher levels of self-management scores in comparison to their counterparts residing in rural areas. This phenomenon may be attributed to the higher level of education exhibited by urban patients, their enhanced ability to acquire health and medical knowledge, and their capacity to comprehend and implement self-management plans more effectively. Furthermore, patients residing in urban areas typically have greater access to medical resources and services, such as healthcare facilities. This has enhanced the awareness and self-management ability of chronic diseases. Conversely, rural areas are characterized by comparatively disadvantaged socio-economic conditions. Patients suffering from chronic diseases may encounter economic pressures and temporal constraints, which can impede their ability to engage in self-management to the fullest extent.

### 3.2 Factor analysis and internal consistency

The validity of the scales was tested by factor analysis, with the samples split into two: one sample was conducted by exploratory factor analysis (EFA) and the other was conducted by confirmatory factor analysis (CFA). The latter was utilized to validate the factor structures of the samples got by EFA. The *T*-test analysis was applied to both samples. The goodness of fit of the scales was assessed by means of the model fit indices (22), supplemented by Maione et al. (23) as: χ^2^/df < 5, RMSEA < 0.08, CFI > 0.90 and NFI > 0.90. Furthermore, Cronbach’s alpha was applied to assess the reliability of the scales. In order to ascertain the validity of the scales utilized in this study, the samples were split into two parts, that is CIRS 1 (*N* = 413) and CIRS 2 (*N* = 413). *T*-test analysis was performed, and no significant difference in group and other variables was observed between the two samples (all *p* > 0.05). An exploratory analysis was conducted to ascertain the factor structure of the CIRS 1. This analysis took the form of a principal component analysis with varimax rotation. The results of this analysis revealed seven principal components, which explained 68.352% of the initial variance (KMO = 0.871, Bartlett’s χ^2^ = 1342.072, *p* < 0.001). The CFA was utilized to process CIRS 2, with the objective of verifying the seven-factor structure of CIRS 1, which had been obtained by EFA. The results of the CFA demonstrated acceptable fit indices (χ^2^/df = 3.65, RMSEA = 0.07, CFI = 0.93, NFI = 0.92). The Cronbach’s alpha for the scale was 0.88. Factor analysis and internal consistency of other scales are detailed in Table 2.

**Table 2:** Factor analysis and internal consistency of scales in this study.

Items	Cronbach’s alpha	Groups *n* = 413	*T*-test	EFA^a^ Principal initial KMO Bartlett’s χ^2^ *p*-value components variance (%)					CFA χ^2^/df RMSEA CFI NFI			
CIRS	0.88	CIRS1	*p* > 0.05	Seven	68.352	0.871	1342.072	<0.001				
		CIRS2							3.65	0.07	0.93	0.92
GSES	0.80	GSES1	*p* > 0.05	One	65.751	0.798	1533.178	<0.001				
		GSES2							4.02	0.07	0.92	0.90
SEHES	0.85	SEHES1	*p* > 0.05	One	70.023	0.825	1724.289	<0.001				
		SEHES2							3.41	0.06	0.95	0.93
CDSMBMS	0.78	CDSMBMS2	*p* > 0.05	Three	72.596	0.814	1,637.344	<0.001				
		CDSMBMS2							3.92	0.06	0.94	0.92

a The analysis was conducted using a principal component analysis with varimax rotation.

### 3.3 Robustness analysis

In order to assess the robustness of this study, the sample was divided into two groups: a male group (*n* = 472) and a female group (*n* = 354). The results are presented in Tables 3, 4. The findings indicate that the aggregate impact of health empowerment on self-management was 0.463 (95% CI 0.342–0.574) for the male group and 0.461 (95% CI 0.350–0.588) for the female group. A range of minor alterations have been identified between the two groups; however, the interpretation of the results remains largely unchanged.

**Table 3:** Bootstrap test for the mediating effect of the chain mediating model for the male group (*n* = 472).

Path	Estimate	LLCI	ULCI	Mediating effect
Health empowerment → chronic disease resource utilization → self-management	0.122	0.060	0.209	26.3%
Health empowerment → self-efficacy → self-management	0.104	0.054	0.145	22.5%
Health empowerment → chronic disease resource utilization → self-efficacy → self-management	0.065	0.029	0.102	14.0%
Direct effect	0.172	0.039	0.312	37.1%
Total effect	0.463	0.342	0.574	100%

**Table 4:** Bootstrap test for the mediating effect of the chain mediating model for the female group (*n* = 354).

Path	Estimate	LLCI	ULCI	Mediating effect
Health empowerment → chronic disease resource utilization → self-management	0.123	0.062	0.207	26.7%
Health empowerment → self-efficacy → self-management	0.104	0.060	0.166	22.6%
Health empowerment → chronic disease resource utilization → self-efficacy → self-management	0.062	0.034	0.106	13.4%
Direct effect	0.172	0.045	0.305	37.3%
Total effect	0.461	0.350	0.588	100%

### 3.4 Scale scores

Table 5 presents the scores for self-management, health empowerment, chronic disease resource utilization and self-efficacy. The self-management score of older adult patients with chronic diseases was found to be (40.89 ± 11.71), with a range of 15–75. The score for health empowerment was (22.07 ± 7.13), with a range of 8–40. The chronic disease resource utilization score was (67.02 ± 10.65), with a range of 21–84. The self-efficacy score was (21.25 ± 4.77), with a range of 10–40. The scores obtained for the domains of self-management, health empowerment, utilization of chronic disease resources and self-efficacy of older adults patients with chronic diseases were all found to be at a medium level.

**Table 5:** Self-management, health empowerment, chronic disease resource utilization and self-efficacy scores (*n* = 826).

Scale and dimension	Score (Mean ± SD)	Z-scores
Self-management	40.89 ± 11.71	
Exercise	13.43 ± 4.23	0.49
Cognitive symptom management	17.78 ± 5.35	0.66
Communication with doctors	9.68 ± 3.42	0.38
Health empowerment	22.07 ± 7.13	
Chronic disease resource utilization	67.02 ± 10.65	
Medical and health teams	12.18 ± 2.78	0.46
Family and friends	9.84 ± 2.66	0.32
Personal responses	11.75 ± 3.07	0.41
Neighborhood communities	10.45 ± 3.15	0.38
Media policies	12.06 ± 4.01	0.45
Organizational structures	10.74 ± 2.09	0.40
Self-efficacy	21.25 ± 4.77	

### 3.5 Bivariate analysis

As demonstrated in Table 6, health empowerment was positively correlated with chronic disease resource utilization (*r* = 0.87, *p* < 0.001) and self-efficacy (*r* = 0.75, *p* < 0.001), as well as self-management (*r* = 0.73, *p* < 0.001). Chronic disease resource utilization was positively correlated with self-efficacy (*r* = 0.68, *p* < 0.001), and self-management (*r* = 0.86, *p* < 0.001). Self-efficacy was positively correlated with self-management (*r* = 0.68, *p* < 0.001).

**Table 6:** Correlation analysis of self-management, health empowerment, chronic disease resource utilization and self-efficacy (*n* = 826).

Item	Chronic illness resources	Medical and health teams	Family and friends	Personal responses	Neighborhood communities	Media policies	Organizational structures	Self-efficacy	Health empowerment	Self-management	Exercise	Cognitive symptom management	Communication with doctors
Chronic illness resources	1.00	0.75^a^	0.68^a^	0.64^a^	0.71^a^	0.65^a^	0.82^a^	0.68^a^	0.87^a^	0.86^a^	0.04	0.09	0.12
Medical and health teams	0.75^a^	1.00	0.89^a^	0.85^a^	0.78^a^	0.92^a^	0.88^a^	0.03	0.03	0.12	0.02	0.02	0.08
Family and friends	0.68^a^	0.89^a^	1.00	0.82^a^	0.79^a^	0.77^a^	0.09	0.10	0.08	0.13	0.03	0.10	0.07
Personal responses	0.64^a^	0.85^a^	0.82^a^	1.00	0.73^a^	0.80^a^	0.04	0.08	0.05	0.09	0.08	0.07	0.09
Neighborhood communities	0.71^a^	0.78^a^	0.79^a^	0.73^a^	1.00	0.82^a^	0.05	0.03	0.05	0.08	0.15	0.10	0.06
Media policies	0.65^a^	0.92^a^	0.77^a^	0.80^a^	0.82^a^	1.00	0.05	0.06	0.02	0.09	0.03	0.06	0.07
Organizational structures	0.82^a^	0.88^a^	0.09	0.04	0.05	0.05	1.00	0.81^a^	0.80^a^	0.86^a^	0.85^a^	0.87^a^	0.84^a^
Self-efficacy	0.68^a^	0.03	0.10	0.08	0.03	0.06	0.81^a^	1.00	0.75^a^	0.68^a^	0.57^a^	0.54^a^	0.56^a^
Health empowerment	0.87^a^	0.03	0.08	0.05	0.05	0.02	0.80^a^	0.75^a^	1.00	0.73^a^	0.67^a^	0.61^a^	0.68^a^
Self-management	0.86^a^	0.12	0.13	0.09	0.08	0.09	0.86^a^	0.68^a^	0.73^a^	1.00	0.72^a^	0.83^a^	0.79^a^
Exercise	0.04	0.02	0.03	0.08	0.15	0.03	0.85^a^	0.57^a^	0.67^a^	0.72^a^	1.00	0.83^a^	0.85^a^
Cognitive symptom management	0.09	0.02	0.10	0.07	0.10	0.06	0.87^a^	0.54^a^	0.61^a^	0.83^a^	0.83^a^	1.00	0.80^a^
Communication with doctors	0.12	0.08	0.07	0.09	0.06	0.07	0.84^a^	0.56^a^	0.68^a^	0.79^a^	0.85^a^	0.80^a^	1.00

a *p* < 0.001.

### 3.6 Mediation effect analysis

The PROCESS program was utilized in order to test the mediating effect of chronic disease resource utilization and self-efficacy on the relationship between health empowerment and self-management. First, health empowerment was designated as the independent variable, self-management as the dependent variable, and chronic disease resource utilization as the mediating variable, with all demographic variables in Table 1 serving as the control variables. The findings indicate that the model exhibits a high degree of compatibility (χ^2^/df = 1.09, CFI = 0.997, TLI = 0.996, RMSEA = 0.014, SRMR = 0.029). As shown in Table 7, the results of the bootstrap test for mediating effect demonstrate that the 95% confidence interval excludes the value of zero, thereby indicating a significant mediating effect. Second, health empowerment was designated as the independent variable, self-management as the dependent variable, and self-efficacy as the mediating variable, with all demographic variables in Table 1 serving as the control variables. The findings indicate that the model exhibits a high degree of compatibility (χ^2^/df = 1.78, CFI = 0.971, TLI = 0.966, RMSEA = 0.044, SRMR = 0.034). As shown in Table 8, the results of the bootstrap test for mediating effect demonstrate that the 95% confidence interval excludes the value of zero, thereby indicating a significant mediating effect. Third, health empowerment was designated as the independent variable, self-management as the dependent variable, chronic disease resource utilization and self-efficacy as the mediating variable, with all demographic variables in Table 1 serving as the control variables. The findings indicate that the model exhibits a high degree of compatibility (χ^2^/df = 1.45, CFI = 0.975, TLI = 0.971, RMSEA = 0.033, SRMR = 0.034). As shown in Table 9, the results of the bootstrap test for mediating effect demonstrate that the 95% confidence interval excludes the value of zero, thereby indicating a significant mediating effect.

**Table 7:** Bootstrap test for the mediating effect of chronic disease resource utilization (*n* = 826).

Path	Estimate	*p*-value	LLCI	ULCI
Health empowerment → self-management	0.275	<0.001	0.148	0.409
Health empowerment → chronic disease resource utilization → self-management	0.185	<0.001	0.120	0.280

**Table 8:** Bootstrap test for the mediating effect of self-efficacy (*n* = 826).

Path	Estimate	*p*-value	LLCI	ULCI
Health empowerment → self-management	0.249	<0.001	0.132	0.382
Health empowerment → self-efficacy → self-management	0.210	<0.001	0.145	0.289

**Table 9:** Bootstrap test for the mediating effect of the chain mediating model (*n* = 826).

Path	Estimate	LLCI	ULCI	Mediating effect
Health empowerment → chronic disease resource utilization → self-management	0.123	0.062	0.207	26.6%
Health empowerment → self-efficacy → self-management	0.103	0.060	0.166	22.2%
Health empowerment → chronic disease resource utilization → self-efficacy → self-management	0.063	0.034	0.106	13.6%
Direct effect	0.174	0.045	0.305	37.6%
Total effect	0.463	0.350	0.588	100%

## 4 Discussion

This study constitutes the inaugural endeavor to examine the relationship between self-management, health empowerment, chronic disease resource utilization, nad self-efficacy among older adult patients with chronic diseases in Southwest China. The study also examines the mediating effect of chronic disease resource utilization and self-efficacy in the relationship between health empowerment and self-management in this population. The study yielded several significant findings.

### 4.1 Self-management, health empowerment, chronic disease resource utilization, and self-efficacy in older adult patients with chronic diseases

Self-management is defined as the process of individuals assuming responsibility for their own health. The concept pertains to the competencies and methodologies employed by individuals to regulate their health and efficaciously manage chronic diseases. These methodologies encompass the monitoring and management of symptoms, the modification of lifestyles, and the engagement in self-care activities aimed at enhancing health conditions (24). Presently, a range of theoretical frameworks underpin the development of self-management behavior patterns. The research on self-management has expanded to encompass all aspects of the process, primarily including planning and goal setting, monitoring and evaluation, self-motivation, learning and improvement, monitoring and adjustment, coping with difficulties, and seeking support. These aspects constitute the fundamental elements of self-management behavior. These techniques have been demonstrated to facilitate enhanced self-regulation and behavioral modification, thereby enabling individuals to achieve their objectives and enhance their overall quality of life. The effective management of oneself requires a combination of knowledge, skills, attitudes and support (25). It is evident that for patients with chronic diseases, effective self-management strategies have the potential to enhance their overall health and well-being. By fostering self-understanding and promoting a comprehensive understanding of their condition, these strategies can contribute to a reduction in the frequency of hospital visits, thereby alleviating the financial and medical pressures experienced by patients and their families (26). The capacity for individuals to self-manage their health conditions has been demonstrated to facilitate recovery and reduce the likelihood of subsequent occurrences (27). The findings of this study indicated that the self-management score of older adult patients afflicted with chronic diseases was moderate (40.89 ± 11.71), a finding that aligns with the outcomes of analogous studies (26–28). The hierarchical arrangement of scores within each dimension, from highest to lowest, is as follows: cognitive symptoms management, exercise and communication with doctors. The reason for the relatively high cognitive symptoms management score might be that the research subjects of this study are recruited from Chengdu, the provincial capital city, which attaches great importance to the popularization of health knowledge among the older adults and the enhancement of their cognitive symptoms management. The low score of exercise in this study may be attributable to the fact that the subjects were older adults people with chronic diseases. The subjects displayed signs of both age-related decline and disease, which appeared to limit their physical capacity and motivation to engage in active behavior. In this study, the communication score with doctors was found to be unsatisfactory. Two potential explanations are posited for this phenomenon. One such factor is the high volume of patients treated by doctors, which results in patients spending less time with their doctor during visits and having limited opportunities for interaction. The second is that some patients, due to the stigma associated with the disease, may be less inclined to proactively engage with healthcare providers and may be less inclined to voice their opinions.

Health empowerment can be defined as a multifaceted approach to enhancing individuals’ awareness of autonomous behavior, alongside a diverse array of social processes aimed at cultivating a conducive environment for healthy living (29). Individuals who can play an active role in their own health can achieve better health outcomes, including better management of chronic diseases, improved mental health and delayed disease progression (30). Moreover, the empowerment of individuals to manage their own health can lead to an increase in their involvement in disease management. This, in turn, can enhance communication with healthcare providers, improve compliance with treatment plans, and ultimately increase patient satisfaction (31). The findings of a research study on the health empowerment status of individuals at risk of kidney disease indicate that enhancing patients’ health perception and social support is conducive to improving their health empowerment level (32). The findings of this study indicated that the empowerment score of older adult patients with chronic diseases was moderate (22.07 ± 7.13), which is consistent with the results of several other studies (33–35). This phenomenon may be attributed to the convivial atmosphere and comprehensive, supportive environment that characteristics the care of the older adults in Chengdu. In this setting, older adults patients afflicted with chronic diseases have unobstructed access to educational resources, including disease prevention and health management training, as well as popular science knowledge and skills. The efficacy of this approach in enhancing patients’ confidence and capacity to make informed self-health decisions has been well-documented, leading to notable improvements in their ability to cope with chronic diseases.

The utilization of chronic disease resources constitutes a sociological approach to the assessment of an individual’s engagement with environmental and social factors. In 2000, Glasgow et al. (36) constructed the social-environmental support pyramid model. The model under consideration is a four-layer pyramid. The initial layer pertains to culture, encompassing media policies and community support, among other factors. The second layer is the environment, which includes the workplace, organizational structure, neighborhood support and physical environment, etc. The third layer is characterized by close relationships, encompassing those among family members, friends, teams, and so forth. The fourth layer is that of individual coping, which encompasses internal factors such as attitude, personality, and beliefs, as well as health management and self-adjustment. This model emphasizes multi-faceted social support and expands the various social resources needed for chronic disease management, which directly affect patients’ self-management. The utilization of chronic disease resources, as viewed through the lens of social ecology, encompasses a multitude of factors across various levels. Primarily, it is employed to evaluate the utilization of diverse social resources by patients afflicted with chronic diseases. The impact of the utilization of chronic disease resources on population health cannot be disregarded. It is evident that the prevalence of chronic diseases is increasing, and there are notable disparities among different regions and populations. In order to assess the quality and effect of the utilization of chronic disease resources, it is necessary to establish a comprehensive and systematic evaluation system that is both scientific and objective. Concomitantly, the utilization of resources is contingent on social and economic conditions, the accessibility of medical services, familial support, and a plethora of other factors. Research has indicated that for patients suffering from chronic diseases, the utilization of social resources has been shown to exert a beneficial effect on the management of their respective diseases (37). Furthermore, the existence of adequate medical resources has been demonstrated to facilitate patient education, thereby enhancing patient compliance, self-care abilities, and overall quality of life (38). The findings of this study demonstrate that the score of chronic disease resource utilization for patients is (67.02 ± 10.65). This indicates that the current situation of resource utilization is relatively ideal and similar to the results of previous studies (8, 9). This may be attributable to the relatively abundant medical resources in the surveyed area. The scores are listed in descending order, with the highest score given to medical and health teams, followed by media policies, personal responses, organization structures, neighborhood communities, and family and friends. In the present study, the highest mean scores were attained by the medical and health teams. This finding suggests that medical staff are the most important resource in older patient disease management, a conclusion that is consistent with the results of many other studies (39, 40). This is due to the fact that, within the context of the doctor-patient relationship, particularly in the case of older adults patients suffering from chronic diseases, medical professionals are able to provide them with the necessary professional medical knowledge and skills to help them effectively manage their chronic diseases. They are also able to offer comprehensive medical evaluations, treatment suggestions and rehabilitation plans, to monitor patients’ health conditions on a regular basis, to adjust treatment plans in a timely manner, and to provide the psychological support and education that patients need. Furthermore, the medical team is able to establish long-term cooperative relationships with older adults patients suffering from chronic diseases, thereby assisting them in formulating reasonable health goals and action plans. These plans are designed to enhance the patients’ self-management abilities and quality of life. In the present study, the lowest score was obtained by the family and friends dimension. This finding suggests that patients perceived a deficiency in the support and assistance they received from their family and friends. This phenomenon may be partially attributed to the cognitive processes and behavior patterns exhibited by older adults patients suffering from chronic ailments. These individuals often adopt a more insular perspective, heavily influenced by the prevailing cultural norms of traditional Chinese medicine and the societal stigmatization associated with illness. Individuals in this demographic often refrain from interacting or communicating with others, driven by a sense of apprehension surrounding the potential for discrimination. This absence of social interaction and support has been shown to impede an individual’s capacity to adapt and to receive assistance from family members, friends and social groups. This, in turn, has a detrimental effect on intimacy and reciprocity within the family unit. The following aspects may be utilized in order to surmount this obstacle: Firstly, the reconstruction of disease cognition is imperative. The popularization of diseases through medical professionals, such as doctors, community health lectures or authoritative medical programs, is a crucial aspect of healthcare promotion. It aims to enhance the comprehension of older adults patients with chronic diseases regarding the scientific understanding of diseases. The objective is to facilitate a shift in perspective, wherein diseases are perceived as an inherent aspect of the natural progression of life, rather than as a personal failure or disgrace. Secondly, the establishment of a support system is crucial in reducing feelings of loneliness. This may be achieved through the formation of peer groups, participation in community chronic disease patient clubs, or online communities such as those found on WeChat. The sharing of experiences within these groups can be a particularly effective method of alleviating feelings of isolation. Furthermore, family meetings should be held to discuss the needs of disease management, with the aim of providing the older adults with a sense of support and understanding, rather than judgment. Thirdly, it is recommended to seek professional assistance in the form of psychological counseling for the older adults and the provision of specialized consultation services for the older adults. Furthermore, the involvement of social work services is recommended. The role of community social workers is to facilitate the connection of older adults patients suffering from chronic diseases with the resources they require, as well as to provide them with emotional support. It is evident that the implementation of the aforementioned measures has the potential to mitigate the impact of traditional Chinese culture and the social stigma associated with diseases on older adults patients suffering from chronic ailments. Furthermore, these measures may also enhance their capacity to cope with their illnesses. The phenomenon of chronically ill older people who are unable to care for themselves often choosing to live with their adult children has been well documented (41). Nevertheless, contemporary Chinese adult children encounter considerable pressure in both their professional and personal lives. A recurrent tendency is for the needs of the next generation to be prioritized over those of the previous generation, which can result in diminished interaction and support with their parents.

Self-efficacy is defined as an individual’s belief in their ability to successfully perform a specific task or activity. It is a substantial element of mental health, underlining the utilization of patients’ inherent motivation to accomplish their desired outcomes (10). A positive sense of self-efficacy has been demonstrated to facilitate a positive approach to disease in patients. Self-efficacy exerts a significant influence on patients’ attitudes toward diseases and health behaviors, consequently impacting the progression of the disease (42). It has been demonstrated that this intervention has the capacity to effect changes in cognition and to rebuild patients’ confidence through health education (43). Patients suffering from chronic diseases who possess a high level of self-efficacy will acquire knowledge related to their diseases through various channels. Furthermore, they will engage in perpetual exploration of their own strengths, accumulation and summarization of their own experience, thereby enhancing their confidence and ability in making health decisions (44). The results of the present study indicated that the mean self-efficacy score of older adult patients suffering from chronic diseases was moderate (21.25 ± 4.77), a result that is comparable to those of several other studies (45, 46). One potential explanation for this phenomenon is that the educational attainment of the respondents is predominantly at the junior high school level or below. This may result in increased cultural and knowledge barriers, which could hinder the respondents’ ability to engage with and comprehend the content. Furthermore, in this study, the older adults may be susceptible to the adverse external environments due to their age and physical condition, which may hinder their ability to adhere to their ideals and goals. Secondly, chronic diseases have a tendency to recur frequently and persist for extended periods of time. Patients are subjected to considerable psychological and physical pressure, which hinders their ability to effectively cope with emotional distress. This, in turn, can lead to a decline in their confidence in the recovery process and potentially impact the efficacy of their recovery.

### 4.2 Correlation analysis of self-management, health empowerment, chronic disease resource utilization, and self-efficacy in older adult patients with chronic diseases

First, the findings of this study demonstrate a positive correlation between the utilization of chronic disease resources and self-management, indicating that the former has a favorable impact on the latter. Consequently, it can be posited that patients who demonstrate higher levels of utilization of available resources tend to exhibit more effective self-management behaviors. This finding aligns with the conclusions drawn from earlier research (47). Research reports (48) indicate that patients’ understanding of their own diseases and treatment plans can be enhanced through attention to medical resources and information, with the result that they are better able to manage and control their diseases. Furthermore, it can assist patients in comprehending and utilizing available medical resources and support, thereby enhancing treatment outcomes and quality of life. As demonstrated in another study (49), a significant correlation has been identified between self-management and family support. The family constitutes the most fundamental social support system for patients, and the utilization of family resources is of great significance for patients’ self-management. The provision of physical and psychological support to patients by family members is of significant benefit, as is the encouragement of continued exercise and the enhancement of self-management skills. The community’s available resources encompass institutions dedicated to community health services, as well as organizations engaged in community volunteer activities. The utilization of these resources enables patients to expand their health knowledge and receive guidance, thereby enhancing their self-management abilities. Concurrently, community resources can offer rehabilitation training, psychological counseling and other services, which are conducive to improving the quality of life and psychological state of patients and promoting the development of self-management behaviors. Furthermore, the positive guidance of media policies, individuals’ coping abilities and a satisfactory working environment provide a robust foundation for self-management. It is therefore vital to study the relationship between self-management behaviors and resource utilization in patients with chronic diseases, in order to gain a more complete understanding of the actual situation regarding the control and treatment of chronic diseases by patients. This will provide a valuable reference for the formulation of more effective medical resource allocation and self-management guidance.

Second, the findings of this study demonstrate a positive correlation between self-efficacy and self-management, indicating that an enhancement in one domain tends to result in a concomitant improvement in the other. Furthermore, the results underscore a favorable impact of self-efficacy on patients’ self-management behaviors. Higher self-efficacy is defined as the belief in one’s ability to execute the behaviors necessary to manage health conditions, and to enhance self-management behaviors. A substantial body of research has demonstrated that patients with high self-efficacy can encourage the adoption and maintenance of healthy behaviors, consequently enhancing their health outcomes (38, 50, 51). Concurrently, effective self-management can also enhance an individual’s sense of self-efficacy, thereby strengthening their confidence and ability in themselves. The findings of a randomized controlled study (52) demonstrate that self-care guided by the TMRM model can enhance patients’ self-efficacy. Within the domain of chronic disease management and rehabilitation, the role of the nursing staff is of particular significance. By fostering self-efficacy, promoting effective self-management behaviors, and facilitating the accumulation of successful self-management experiences, these professionals can empower older adults patients. This, in turn, can contribute to the formation of a positive cycle, thereby enhancing self-efficacy and enabling more effective control of chronic diseases, ultimately leading to an improvement in quality of life among older adults patients with chronic diseases.

Third, the findings of this study demonstrate a positive correlation between health empowerment and self-management, indicating that an enhancement in health empowerment is associated with an improvement in self-management behavior. This observation is in alignment with the previous research findings on patients with type 2 diabetes (53). Flournoy-Floyd et al. (54) intervened with patients suffering from systemic lupus erythematosus based on the empowerment, incentive and education model. The results demonstrated that it could reduce the disease activity and fatigue levels of patients, improve their self-efficacy and treatment compliance, and promote the formation of healthy behaviors. It is evident that health empowerment has the capacity to enhance an individual’s sense of self-efficacy, thereby engendering a sense of confidence and capability in regard to self-management.

Participation in decision-making, the formulation of plans and goals, and the acquisition of health information and resources are pivotal in older patients with chronic diseases to develop a comprehensive understanding of their health conditions and to enhance their self-management abilities. This, in turn, self-management has the capacity to facilitate the realization of health empowerment. It is evident that patients are able to develop a more precise understanding of their health conditions through self-monitoring, self-assessment and self-feedback. Consequently, they become more capable and confident in participating in health decision-making and achieving health empowerment. For older patients suffering from chronic diseases who require self-management, enhancing their sense of health empowerment, encouraging active participation in the healthcare process, and enabling them to grasp their own health conditions and decision-making power can help improve the effectiveness of disease management and their quality of life.

### 4.3 The mediating effects of chronic disease resource utilization and self-efficacy between health empowerment and self-management in older adult patients with chronic diseases

First, the findings of this study demonstrate that the utilization of chronic disease resources exerts a partial mediating effect between health empowerment and self-management, with the mediating effect accounting for 26.6%. The concept of health empowerment has the capacity to exert a direct and positive influence on self-management. Moreover, it can exert an indirect influence on self-management through the utilization of resources associated with chronic diseases. It is widely accepted that health empowerment constitutes the basis for individuals to effectively self-manage chronic diseases. It is imperative to empower individuals and allocate resources with the objective of enabling them to better understand and manage their own health conditions. As demonstrated in research (55), individuals who possess a heightened sense of health empowerment are more inclined to adopt positive self-management behaviors. The utilization of chronic disease resources, as an external environmental factor within the theoretical framework of health empowerment, has the potential to garner comprehensive external support through the active leveraging of social resources. When patients possess the relevant knowledge, skills and resources, they are better equipped to understand the characteristics and management needs of chronic diseases and can adopt a more proactive approach to self-management. The acquisition of accurate health information, the assimilation of effective self-management strategies, and the mastery of health monitoring tools empower patients to formulate and implement treatment plans autonomously, and to adopt positive health behaviors. The utilization of chronic disease resources functions as a conduit, thereby facilitating patient access to essential health information, professional counsel, and support services. This, in turn, fosters enhanced patient confidence and capacity for self-management. Consequently, by empowering patients to manage their diseases, they can optimize the utilization of chronic disease resources and thereby enhance their self-management behaviors. The utilization of chronic disease resources has been demonstrated to provide patients with knowledge and resources, thereby enabling them to enhance their ability to cope with and manage chronic diseases and to improve their health conditions.

Second, the findings of this study demonstrate that self-efficacy exerts a partial mediating effect between health empowerment and self-management, with the mediating effect accounting for 22.2%. The concept of health empowerment has the capacity to exert a direct and positive influence on self-management. In addition, it may exert an indirect influence on self-management through the medium of self-efficacy. In accordance with Bandura’s self-efficacy theory (17), a patient’s confidence in their ability to successfully carry out a specific behavior will directly affect their self-management behavior. Consequently, patients who perceive themselves to possess the necessary capabilities for self-managing chronic diseases are more inclined to adopt such active strategies. This finding aligns with the results of previous research (56), which demonstrated a significant positive association between self-efficacy and patients’ self-management behaviors. It is imperative to exert influence on patients in order to effect changes in their self-management behaviors; this can be achieved by enhancing their sense of self-efficacy. For instance, following the provision of support and assistance from medical staff, patients will have greater confidence and motivation to proactively manage their disease. As demonstrated in another study (57), health empowerment has been shown to enhance the quality of life of patients. Among the factors identified, patients’ confidence in treatment and peer support have been shown to be significant in enhancing the quality of life. This, in turn, has been demonstrated to promote the improvement of self-management behaviors by enhancing patients’ self-efficacy. The direct impact of health empowerment on self-management can facilitate the design of effective health intervention strategies. The provision of relevant health knowledge and skills is an essential component of this approach, as is the enhancement of patients’ self-management abilities and the encouragement of proactive actions to manage chronic diseases. This includes the regular organization of educational and training activities, the establishment of support networks and community resources, and the improvement of accessibility and quality of medical services. The implementation of these intervention measures has been demonstrated to facilitate enhanced patient comprehension and proficiency in chronic disease management, thereby fostering increased engagement in self-directed healthcare.

Third, the findings of this study demonstrate that the utilization of chronic disease resources and self-efficacy act as chain mediating factors, thereby linking health empowerment and self-management. The chain mediating effect accounts for 13.6% of the observed outcomes. The present study established that the utilization of resources dedicated to chronic diseases has a positive impact on self-efficacy. The utilization of chronic disease resources is defined as an individual’s capacity to procure and employ medical and health resources pertaining to chronic diseases. The effective utilization of these resources has the potential to enhance patients’ confidence in the management of chronic diseases. The enhancement of self-efficacy has been demonstrated to encourage individuals to engage more proactively in self-management behaviors, adopt effective control strategies and modify their lifestyles (58). This transformation of self-efficacy is predicated on an individual’s belief and expectation that they can effectively utilize chronic disease resources for self-management. Chronic diseases are defined as chronic conditions requiring long-term treatment and management. In order to enhance their sense of self-efficacy and improve their ability to control their health conditions, patients must make rational use of a variety of medical and social resources during the treatment process. The promotion of health empowerment and self-management are pivotal components in the treatment and management of chronic diseases, with the potential to assist patients in enhancing their ability to effectively control their health conditions. Consequently, the utilization of chronic disease resources exerts an indirect influence on individuals’ self-management behaviors by enhancing their sense of self-efficacy.

### 4.4 Implications for future interventions

Firstly, medical staff should provide health education and training for older adults patients suffering from chronic diseases. This will help them to correctly understand and rationally utilize medical and community resources, thus improving their utilization of said resources. Secondly, the establishment of a comprehensive social support platform under the auspices of the government is recommended. This platform should be tasked with the provision of psychological support, health education, and popular science on scientific disease management for older adults patients suffering from chronic diseases. The platform should aim to correct the patients’ cognition and beliefs, build their confidence and courage, and thereby enhance their sense of self-efficacy. Secondly, the establishment of an effective communication mechanism between older adults patients suffering from chronic diseases and medical professionals, as well as their family members, is paramount. The empowerment of these individuals through education and guidance is of the utmost importance, encompassing the inculcation of accurate health concepts and lifestyles. The formulation of personalized health management plans is essential, ensuring the participation of these individuals in health management and decision-making processes. The enhancement of their health empowerment level is crucial, as is the improvement of patients’ self-management abilities.

The fundamental principle underpinning all intervention measures is the respect for the autonomy, experience and value of older adults patients suffering from chronic diseases. The concept of health empowerment does not entail the mere “grants” of power to patients; rather, it is an approach that fosters the inner capabilities and self-assurance of individuals, thereby engendering a sense of autonomy in their health management. The empowerment and self-management of older adults diabetic patients can be taken as a case study. Firstly, it is crucial to empower patients with the necessary knowledge. Nurses can utilize food models to instruct patients in the estimation of carbohydrate content, as opposed to merely providing a list of prohibited foods. Secondly, the utilization of technology to empower patients is advocated, with the objective of facilitating the comprehension and practical application of methodologies such as the use of a rudimentary blood glucose meter and the maintenance of a blood glucose diary. This diary should encompass the patient’s dietary intake and physical activities during the specified time period. Secondly, the empowerment of patients in decision-making processes is paramount. A collaborative approach is adopted whereby doctors and patients review blood sugar records together, and make decisions as a result of the discussion. “It was observed that blood sugar levels exhibited a marked increase following the ingestion of porridge. The proposal is to substitute whole wheat bread and eggs for breakfast.” In addition, it is imperative to furnish patients with assistance and a sense of empowerment. Patients should be invited to participate in a diabetes patient group, in which they can engage in morning exercises alongside others, exchange recipes, and share experiences of effective blood sugar management. Finally, it is essential to provide environmental empowerment for patients: The establishment of “low-sugar food zones” within healthcare facilities and communal shared spaces is recommended, with the objective of facilitating enhanced comprehension among older adults patients regarding the foods that are conducive to their health and well-being. The implementation of these measures has been demonstrated to empower older adults patients suffering from chronic diseases, enabling them to assume a position of mastery over their own health. This mastery is characterized by the acquisition of knowledge, the development of confidence, the utilization of effective tools, and the provision of comprehensive support. The outcome of these efforts is the enhancement of patients’ self-management abilities in the context of chronic diseases.

### 4.5 Limitations

This study has several limitations. First, the cross-sectional design of the present study has the potential to introduce bias, and the necessity for longitudinal studies to confirm the findings is evident. The subsequent research plan is as follows: The selection of research subjects will be conducted through the utilization of standard sampling methods, with the objective of recruiting 100 patients afflicted with chronic diseases from tertiary grade A hospitals in Chengdu. The collection of data will be achieved through the utilization of semi-structured interviews, with the subsequent analysis being conducted via thematic analysis. This approach is designed to facilitate the generation of explanatory insights into the underlying causal mechanisms. Second, the present study was conducted in 12 tertiary hospitals in Sichuan; therefore, the generalisability of the findings to different levels of hospitals in other provinces should be considered with caution. Third, in subsequent studies, it would be advisable to give further consideration to the factors that influence older patients suffering from chronic diseases. Such factors may include psychological resilience, disease burden and disability other variables. The aim of this would be to enhance the research model. The subsequent research plan is as follows: In light of the findings of this study, it is proposed that the incorporation of further variables be undertaken, including psychological resilience, disease burden and disability. The objective of this incorporation is to facilitate the exploration of the relationships among these variables. For instance, the establishment of a mixed model with moderating mediators among these variables is a potential avenue for future investigation.

## 5 Conclusion

The present study found that the patients exhibited medium levels of self-management behaviors. The utilization of chronic disease resources, self-efficacy and health empowerment of older adults patients with chronic diseases are all positively correlated with self-management. The better the empowerment of older adults patients with chronic diseases, the better they will utilize resources, and the higher their self-efficacy will be. This will lead to better self-management behavior for chronic diseases. Furthermore, the concept of health empowerment has been demonstrated to exert a direct influence on self-management practices. Additionally, it has been observed that health empowerment can indirectly impact self-management through the utilization of resources specific to chronic diseases and the enhancement of self-efficacy. The utilization of chronic disease resources, in conjunction with self-efficacy, has been demonstrated to serve as a mediating factor between health empowerment and self-management.

## Data Availability

The original contributions presented in the study are included in the article/supplementary material, further inquiries can be directed to the corresponding author.
